# A Perspective on Bio-Inspired Approaches as Sustainable Proxy Towards an Accelerated Net Zero Emission Energy Transition

**DOI:** 10.3390/biomimetics10120842

**Published:** 2025-12-16

**Authors:** Miguel Chen Austin, Katherine Chung-Camargo

**Affiliations:** 1 Centro de Investigación e Innovación Eléctrica, Mecánica y de la Industria (CINEMI), Universidad Tecnológica de Panama, Panama City 0819-07289, Panama; 2 Research Group Energy and Comfort in Bioclimatic Buildings (ECEB), Faculty of Mechanical Engineering, Universidad Tecnológica de Panama, Panama City 0819-07289, Panama; katherine.chung@utp.ac.pa; 3 Sistema Nacional de Investigación (SNI) AIP, Panama City 0816-02852, Panama

**Keywords:** biomimicry, energy transition, net zero emissions, renewable energy systems, readiness level

## Abstract

The global energy transition faces a chasm between current policy commitments (IEA’s STEPS) and the deep, rapid transformation required to realize all national net zero pledges (IEA’s APC). This perspective addresses the critical innovation and policy gap blocking the APC pathway, where many high-impact, clean technologies remain at low-to-medium Technology Readiness Levels (TRLs 3–6) and lack formal policy support. The insufficient nature of current climate policy nomenclature is highlighted, which often limits Nature-based Solutions (NbS) to incremental projects rather than driving systemic technological change (Bio-inspiration). Then, we propose that a deliberate shift from simple biomimetics (mimicking form) to biomimicry (emulating life cycle sustainability) is the essential proxy for acceleration. Biomimicry inherently targets the grand challenges of resilience, resource efficiency, and multi-functionality that carbon-centric metrics fail to capture. To institutionalize this change, we advocate for the mandatory integration of bio-inspired design into National Determined Contributions (NDCs) by reframing NbS as Nature-based Innovation (NbI) and introducing novel quantitative metrics. Finally, a three-step roadmap to guide this systemic shift is presented, from deployment of prototypes (2025–2028), to scaling evidence and standardization (2029–2035), to consolidation and regenerative integration (2036–2050). Formalizing these principles through policy will de-risk investment, mandate greater R&D rigor, and ensure that the next generation of energy infrastructure is not just carbon-neutral, but truly regenerative, aligning technology deployment with the necessary speed and depth of the APC scenario.

## 1. Introduction

Despite the growing interest in nature-based and bio-inspired solutions for energy systems, existing research remains fragmented across technological, ecological, and socio-technical domains.

Current reviews focus on individual components—such as biomimetic photovoltaic structures, adaptive cooling systems, or bio-inspired materials—yet none integrate these developments within the broader context of net zero transition pathways (e.g., STEPS/APC).

Moreover, prior work has not systematically assessed the maturity or applicability of biomimetic innovations using validation levels or TRL indicators, nor has it distinguished between biomimicry, biomimetics, and others in a reproducible way. Frameworks linking Nature-inspired solutions to energy transitions exist, but they lack operational translations to socio-technical transition mechanisms such as Common Boundary Concepts (CBCs), and they generally overlook policy instruments and readiness constraints.

Thus, this perspective addresses these gaps by:

i. Synthesizing bio-inspired energy solutions across generation, storage, and management domains.
ii. Introducing a reproducible codebook for classifying biomimetic approaches.
iii. Evaluating their readiness using TRL and validation criteria.
iv. Connecting biological principles to socio-technical CBCs.
v. Outlining a coherent, policy-oriented roadmap for how biomimicry can contribute to achieving national net zero pledges under an APC-aligned pathway.

This integrated framing provides not a traditional review, but a forward-looking perspective on the transformative role of bio-inspired design in the energy sector transition.

However, this transition faces significant hurdles: many high-impact, innovative energy technologies remain at low Technology Readiness Levels (TRLs 3–6), and the current global policy nomenclature (such as NDCs) exhibits a distinct absence of explicit, formal recognition for advanced methodologies like biomimetics or biomimicry. In contrast, this perspective paper argues that bio-inspired design, encompassing both structural-based biomimetics and principles-based biomimicry, offers a powerful methodology to accelerate this required innovation.

The realization of the ambitious net zero emission (NZE) targets, as outlined in the IEA’s Announced Pledges Case (APC), is fundamentally obstructed by the low Technological Readiness Levels (TRLs 3–6) of key innovations and a policy gap within global climate commitments (NDCs). Bridging this gap requires a mandatory, systemic transition from pure biomimetics (mimicking form/function) to biomimicry (emulating life cycle sustainability and regeneration), coupled with the formal integration of bio-inspired principles into national policy frameworks to accelerate technological development and ensure a truly sustainable and regenerative energy system.

**Figure 1:**
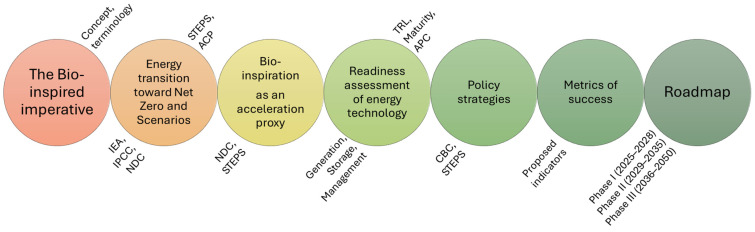
Flowchart showing all the steps and points that you have covered in this work: Starting with why considering nature as a reference, follow by bio-inspiration as a proxy toward accelerating NDC robustness and technology innovation, and then a proposed roadmap.

The content is structured to first establish the bio-inspired imperative by defining the critical difference between structural-based biomimetics and principles-based biomimicry (Figure 1). The subsequent section assesses the readiness of current bio-inspired energy systems for deployment under the ambitious APC scenario, highlighting the low Technological Readiness Levels (TRLs 3–6) and their potential impact across generation, storage, and management. This analysis is then leveraged to propose a set of policy recommendations aimed at formally integrating bio-inspired design into national climate action (NDCs). Finally, the manuscript provides a roadmap for transitioning to truly regenerative energy systems, proposing novel, quantitative metrics of success (such as FPI and LCSI) to validate regenerative outcomes beyond purely carbon-centric measurements.

## 2. The Bio-Inspired Imperative

Nature defines success as maintaining favorable conditions to sustain life over several generations by upcycling everything [1]. Thus, focusing on reducing carbon emissions alone limits real success.

**Figure A1:**
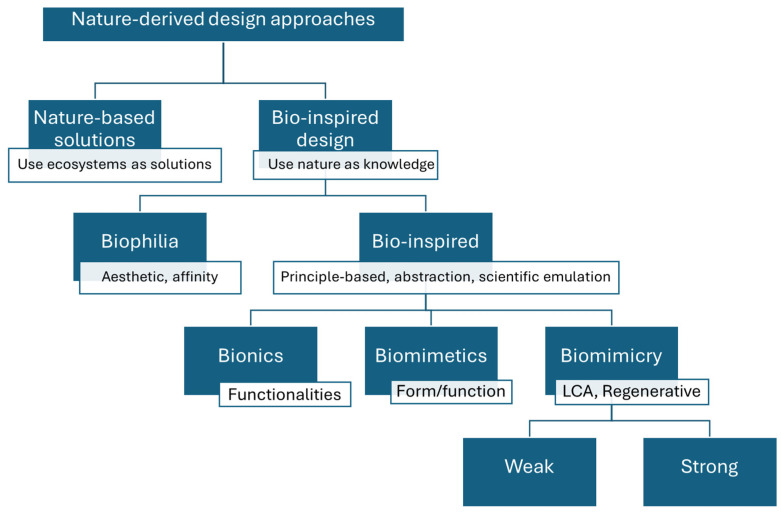
Schematic hierarchy of nature-derived design approaches to help understand the proposed codebook for bio-inspired design concepts and terminology.

The energy-oriented research literature indicates that the main reason researchers look at nature for inspiration is to develop more sustainable, cleaner, environment-friendly ways of harvesting energy (in other words, to apply “strong” biomimicry, which assumes nature’s perfection or at least superiority over human design [2,3]). From here on out, concepts’ definitions follow Table A2 and Figure A1).

The principal paradigm that leads bio-inspiration to evolve into applicable strategies is translating the possibilities offered by natural design, i.e., increased resilience, multi-functionality, and lower degree of risk, into a higher sustainability [4]. This is what natural energy harvesting is supposed to offer.

However, most studies are presumed to implement mimicking approaches rather than the biomimicry approach (either via the problem-based, solution-based, or their combination, i.e., spiral) to contextualize such inspiration.

The importance of distinguishing both concepts lies in the willingness researchers exhibit to develop solutions inspired by nature, but the focus on satisfying human needs limits innovation toward sustainability. This is defined as weak biomimicry, which assumes nature is still under development, and hence might not supply the solution to any problem; hence, natural solutions need to undergo scrutiny under the sustainability criteria [2].

This is worth mentioning because the interchangeability of both terms is still mistakenly employed among researchers, architects, and designers. A clear and straightforward analysis of similarities and differences can be found in [5,6]. A clear and straightforward example lies in the need to develop more efficient solar cells/panels, either by avoiding overheating via sweat-promoting gels [7] or allowing self-cleaning via the lotus effect [8], where

- The biological functionality is successfully transferred (biomimetics) using conventional manufacturing processes (with high pressure and high temperature), and alloys with either non-local or toxic materials.
- Or the biological functionality is successfully transferred together with low-negative environmental impact manufacturing processes and local non-toxic materials, considering and leveraging its life cycle and end of life (biomimicry).

Finally, since such bio-inspiration may not always come directly from entire organisms or ecosystems, e.g., ears, muscles, organs, the literature coins the term “pinnacle” to refer to all.

With the no-use of the terms “biomimicry” and “biomimetics-”, the prefix “bio” is frequently spotted in recent documents, which actually is intended to be referred to from the same perspective as “inspired by nature-”. This indicates that cautiousness is still prompted to use the most meaningful terms when asking to what extent you are letting the design be inspired by nature: “biomimicry” or “biomimetics.” Attention should be paid to this issue since a design solution which is “biologically inspired” does not intrinsically imply that the design solution is “nature-based” because the former is embedded within the definitions of nature-based solutions [9]. Nature-based solutions need a precise understanding of socio-technical regimes far from other types of innovation [9,10].

Although the mere mimicking of natural mechanisms, i.e., biomimetics, may be enough for some applications such as enhancing vehicles’ aerodynamics via the modifi-cation of geometry (how they look) and materials (what they are made of), cooling [11] and heating [12] systems, and the advancement of energy systems for harvesting and generating, crave for the need of grasping the biological processes and functions implied. However, this process needs to also go further and plan for the sustainability, i.e., biomimicry [1], of such energy systems.

In addition, the use of the term “biomimetic system” was recently coined by the International Standard Organization (ISO) 18458:2015 (recently reviewed and confirmed in 2021) [13], which claims, “If a technical system is subjected to a development process according to this International Standard, then it is allowed to be referred to as a ‘biomimetic’ system” [13].

## 3. The Typical Need to Focus on Energy Transition to Reach Net Zero Emissions

Climate change challenges are said to be essentially based on energy, because energy demanding sectors (generation, industry, transportation, buildings, etc.) cause about 68% of global greenhouse gas (GHG) emissions [14], which means that a CO_2_ equivalent neutral production of energy must be at the center of any solution [15].

The Global Industry Classification Standard (GICS) defines the energy sector as the sector that comprises companies’ businesses dominated by the following: (i) the construction or provision of oil rigs, (ii) drilling equipment, and (iii) other energy-related services and equipment. Such companies engage in the exploration, production, marketing, refining, and transportation of oil-based and gas-based products [16].

The energy sector is currently focusing efforts to reduce fossil fuels usage (decarbonization), strengthening the transition to renewable sources for distributed energy generation and non-toxic energy storage systems (decentralization) [17], and optimizing energy management (digitalization) [17,18]. However, decarbonization may be the most critical aspect in this sector [19].

**Figure 2:**
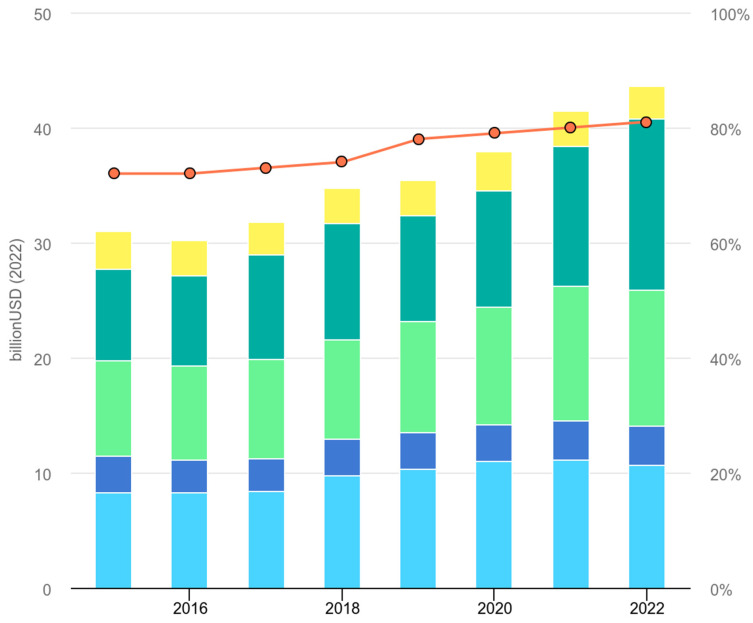
Investment trend on energy system research and development by governments from 2015 to 2022: North America (light blue); Japan, Korea, Australia, and New Zealand (blue); Europe (light green); Chine (green); Rest of the World (yellow); Share of clean energy. Taken from [21] under the license Creative Commons Attribution 4.0 International (CC BY 4.0).

Behind these three major challenges, a few important aspects that may need deeper consideration to tackle those mentioned above can be evoked. For instance, the energy transition will require higher energy investment; back in 2022, a growth of about 4% investment was said to be needed to be reached to support such an energy transition [20]. Gathered data, up to 2022, showed an increasing trend of Governments investment toward research and development (Figure 2), where northern governments are investment leaders [21], which presents a promising pathway towards the net zero target since China holds the majority of emissions [20] from the industrial sector.

Although said investment growth will help emerging new technologies driven by the decarbonization and digitalization ambition, the “niche” energy technologies [19], e.g., CCUS, will need to turn to “mainstream” [20], making them affordable to successfully decentralize their usage, resulting in a financial challenge [10,15,19,22,23]. This will require the rapid development of technologies, mostly still in the early stages. In 2020, a market assessment by the IEA showed the need for the readiness of 400 technologies, of which only half were said to be available back in 2020, required for additional emissions savings to reach the net zero emissions target by 2050 [15].

Around 60% of pledges aim to achieve net zero emissions by 2050, but several companies have set an earlier deadline of 2030 or 2040 [24]. Such a net zero target requires a substantial acceleration in the transitions to a full life cycle clean and sustainable energy that is already underway in many countries and industries [15]. There has been a rapid increase in governments making pledges to reduce GHG emissions to net zero.

**Figure 3:**
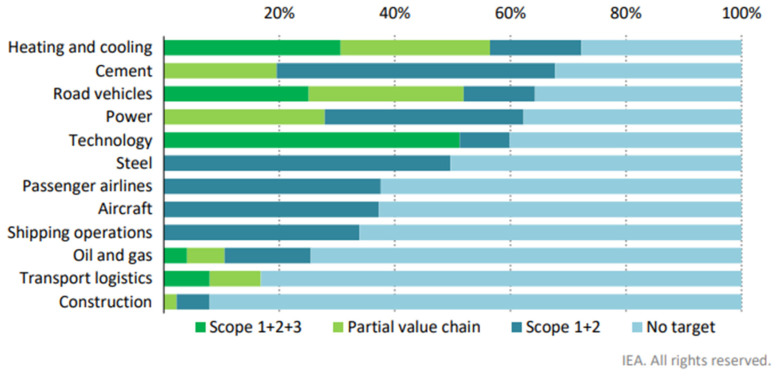
Sectoral activity of large energy-related companies with announced pledges to reach net zero emissions by 2050. Direct emissions from energy and other sources owned or controlled (scope 1). Indirect emissions from the production of electricity and heat, and fuels purchased and used (scope 2). Indirect emissions from sources not owned or directly controlled but related to their activities (scope 3). Taken from [24], under the license Creative Commons Attribution 4.0 International (CC BY 4.0).

Global oil demand summit and gas demand growth are expected to increase by 10% in the next few years and cope by 2035 [20], leading to severe economic effects due to the tremendous amount of carbon-fuel-running devices in the transportation sector and industries, and the space and energy needed to discard said devices for recycling or waste; they are more so to the carbon-fuel-running energy systems in massive industry sectors such as shipping (boats), civil aviation (airlines), and construction (Figure 3). Although, company pledges may not be readily comparable due to a wide variability in coverage and timeframe [24], such industries risk trouble in reducing carbon emissions [15] and will face scrutiny [23].

The use of offsets could be a cost-effective mechanism to eliminate emissions from parts of value chains where emissions reductions are most challenging, provided that schemes to generate emissions credits result in permanent, additional and verified emissions reductions. However, there is likely to be a limited supply of emissions credits consistent with net zero emissions globally and the use of such credits could divert investment from options that enable direct emissions reductions [24].

All the above-mentioned evidence shows that one part of the problem lays in providing the funding for technological development for reduced carbon emissions energy systems and the other part lays in the carbon emissions measurement capacity, as this limits accurate balanced compensation. Either way, focusing only on the energy transition emissions to more clean energy power generation will not be enough, because a great part of the problem also lies in energy demand. “Clean power is not yet outrunning global electricity demand growth” [25], but increasing efforts are currently being put into the digital infrastructure through higher investment than to fossil fuel industry [26].

## 4. Energy Transition Framed in NZE Scenarios and Actions

The world 2050 NZE goal requirements are focused on emissions. Reducing emissions alone may have a positive impact on the quality of life of many people worldwide since industry holds a tremendous number of low-income workers. However, the inclusion of measure-to-success is needed to verify the impact of technology, putting the environment and society up front.

A report on a roadmap for the global energy sector toward achieving a scenario of net zero emissions (NZE) by 2050 was published by the International Energy Agency (IEA) [24]. As forecasted, such a scenario comprises the following:

- Renewables reach almost 90% of total electricity generation.
- Nearly 70% of electricity generation globally from solar photovoltaic (PV) and wind.
- More than 85% of buildings are zero-carbon ready.
- More than 90% of heavy industrial production is low emissions.
- A total of around 7.6 Gt CO_2_ is captured.
- A total of around 0.52 Gt CO_2_ is from low-carbon hydrogen.

Such a scenario is driven by two proposals, the Stated Policies Scenario (STEPS) and the Announced Pledges Case (APC) [24]. The IEA’s STEPS concerns the consequences of existing and stated policies for the energy sector. It draws on the latest information on national energy and climate plans and the policies underpinning them. It takes account of all policies backed by robust implementing legislation or regulatory measures, including the Nationally Determined Contributions (NDCs) that countries have put forward under the Paris Agreement up to September 2020, and the energy components of announced economic stimulus and recovery packages.

So far, few net zero emissions pledges have been backed up by detailed policies, implementation plans, or interim targets: most net zero pledges are not included in the STEPS. On the other hand, the APC works under the assumption that all national net zero emissions pledges are fully realized and on time. It, therefore, goes beyond the policy commitments incorporated in the STEPS. The APC aims to see how far the full implementation of the national net zero emissions pledges would take the world towards net zero emissions and to examine the scale of the energy sector’s transformation that such a path would require [24].

**Figure 4:**
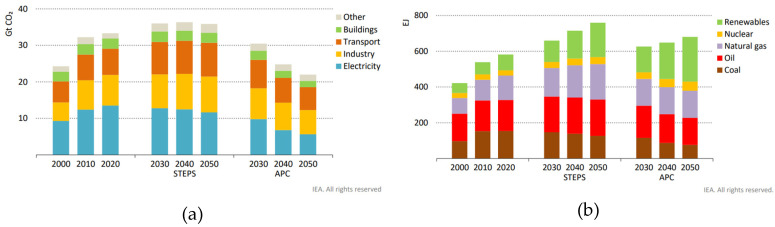
Forecasted STEPS and APC NZE 2050 scenarios by: (**a**) global CO_2_ emissions by sector and (**b**) total energy supply, proposed back in 2021. Taken from [24] under the license Creative Commons Attribution 4.0 International (CC BY 4.0).

The STEPS announced that net zero pledges would cut emissions in 2050 by 60% in the electricity sector, 40% in buildings, 25% in industry, and just over 10% in transport. Net zero pledges to lift renewables in the APC from 12% of the total energy supply in 2020 to 35% in 2050 may be achieved mainly at the expense of coal and oil [24]. As such, the global emissions by sector and the total energy supply by source, following STEPS and APC NZE scenarios, are presented in Figure 4.

The pledge approach implemented in the APC presents the essential implications for the energy system. A net zero pledge for all emissions does not necessarily mean that the energy sector’s CO_2_ emissions need to reach net zero. For instance, countries’ net zero plans may anticipate that residual energy-related emissions are compensated by the absorption of emissions from forestry or land use or by harmful emissions arising from bioenergy or direct CO_2_ from the air with carbon capture, usage, and storage (CCUS). It is impossible to know precisely how net zero pledges will be implemented. Still, the design of the APC, particularly concerning the energy system pathway, has been informed that many national bodies have developed to support net zero pledges. Countries’ policies still having not made a net zero pledge, including population and economic growth, are assumed under the STEPS proposal scenario [24].

Therefore, the near future is trending towards a mixed net zero scenario rather than inclined to either STEPS or APC. However, comparing both proposal scenarios STEPS and APC (Figure 4) shows that the ideal pathway to follow is the APC. This implicates the faster development of CCUS and innovative energy generation technologies for a sustainable energy transition, while improving and increasing the ambition of net zero pledges through NDCs.

## 5. Bio-Inspiration as an Acceleration Proxy Towards an Integrated NZE Future

NDCs must be updated every five years with progressively higher ambition [27]. The current cycle, resulting in the 2025 NDC Synthesis Report, confirms a pivotal transition in climate governance [28]. The shift is characterized by a move beyond mere mitigation targets to integrating adaptation, finance, technology transfer, and capacity-building across all dimensions of climate action [29].

The report documents that NDCs are evolving into comprehensive, “whole-of-government, whole-of-economy, and whole-of-society instruments” [28].

This implementation imperative demands a focus on quality, credibility, and expanded economic coverage, evidenced by 89% of new parties communicating economy-wide targets [29].

Critically, the need for “strong, ongoing international cooperation” and “new and innovative approaches to unlock finance and support” is explicitly highlighted as essential for implementing the new NDCs at scale [29].

These innovative approaches must deliver solutions that are highly efficient, inherently resilient, and fundamentally regenerative, pointing toward disciplines that transcend conventional engineering.

The integration of bio-inspired design, encompassing biomimicry and biomimetics, offers a powerful, scientifically validated methodology to meet this demand for systemic innovation. Recent research projects are concerned with the coupling of nature-based solutions [10], showing that exposure to biological examples increases novelty in design ideas, while exposure to human-engineered examples limits variety in design ideas [30].

Nevertheless, official NDC policy documents, including the NDC registry, the primary UNFCCC NDC resources, and the 2025 NDC Synthesis Report, indicate a distinct absence of explicit terminology such as “biomimicry”, “bionics” or “biomimetics”. The current policy nomenclature focuses on established, broader categories: adaptation, technology transfer, and capacity building [29].

The lack of direct reference suggests a strategic disparity between the stated necessity for radical innovation and the language used in high-stakes political documents like NDCs. NDCs prioritize established, quantifiable strategies, such as afforestation, solar energy deployment, and methane reduction, over emergent or complex innovation methodologies that currently lack unified global metrics. This inherent conservatism in policy documentation risks failing to fully leverage the potential for systemic change offered by bio-inspired innovation.

The current evidence suggests that where bio-inspired design is deployed (e.g., in regenerative architecture or specific educational initiatives), it is often driven by individual stakeholders, local authorities, or academic consortia, as observed in initiatives across France [31], China and Panama [32,33], among others. The ambitious targets set by NDCs necessitate the principles inherent in bio-inspired design, such as breakthroughs in material science and system optimization that biomimetics and bionics are poised to deliver.

The localized, fragmented nature of this application demonstrates a gap: national NDCs have not yet moved to institutionalize this innovative approach across whole-of-government planning.

Similarly, the fact that 73% of NDCs now feature adaptation and resilience demands methodologies, like those offered by ecosystem-level biomimicry, can integrate ecological integrity into engineered systems.

The challenge, therefore, is to move these advanced, nature-validated concepts from sub-national experimentation into the formalized national policy mandates of future NDCs, such as the documentation proposed in 2024 for NDCs 2025 [34]. However, adopting bio-inspired technologies requires significant industrial change, particularly in supply chains, workforce training, and infrastructure [35]. Here, to minimize risks, pilot programs or phased implementation could be introduced, allowing industries to adapt for successful integration. This adoption depends on market readiness, policy frameworks, public perception, and economic feasibility, with long-term benefits such as job creation and GDP growth potentially outweighing initial high costs [35].

Such innovative technology employing mechanical parts and control systems may suffice today’s necessity of lowering GHG emissions. However, large-scale electrical energy storage and retrieval will almost certainly be required to raise renewable sources’ penetration into the grid [36]. Understanding this coupling between biomimetics/biomimicry and energy system technologies requires exploring theoretical foundations and conceptual linkages [10] and a multidisciplinary team of professionals who can increase the readiness of such new technologies by highlighting the key aspects of nature’s involvement.

## 6. Readiness Assessment of Energy Technology Developments to Foster an APC Scenario Pathway

The technologies compiled in the revised Table A3 illustrate a spectrum of biomimetic- and biomimicry-based innovations targeting energy generation, storage, and management systems. When evaluated through the lens of the IEA Announced Pledges Case (APC) scenario, these developments reveal promising but uneven readiness levels for large-scale deployment.

(a) Technological readiness and validation context

Most of the technologies fall between TRLs 3 and 6, corresponding to concept-to-prototype stages validated primarily in laboratory settings. This indicates that while the scientific foundations of nature-inspired design (e.g., self-cleaning lotus-inspired PV films, moth-eye antireflective surfaces, or evaporative “sweat” cooling gels) are strong, industrial scalability and durability validation remain limited. In the APC context, this stage is insufficient for rapid emission mitigation, which depends on TRLs 8–9 technologies ready for commercialization within the 2030–2040 window. Nevertheless, their high potential for efficiency gains and passive performance optimization suggest that these systems could complement mature renewables by reducing embodied energy and maintenance requirements.

(b) Alignment with APC transformation pillars

The APC scenario emphasizes deep electrification, accelerated renewable deployment, and efficiency improvements across all sectors. Biomimetic energy generation approaches—particularly those enhancing solar energy capture and thermal regulation—are directly aligned with these pillars. For example, the leaf-mimic PV devices and photosynthetic light-trapping layers demonstrate up to +55% electricity output improvements, supporting APC’s target of doubling clean power generation by 2040. Similarly, cooling strategies inspired by sweat evaporation enhance the operational efficiency of PV systems under high-temperature tropical conditions, extending asset lifespans—a key factor in reducing life cycle emissions. These incremental innovations, though currently low-TRL, could become pivotal in region-specific decarbonization strategies, especially in the Global South, where climatic resilience and material adaptability are essential.

(c) Integration barriers and enabling mechanisms

To reach the APC’s system-wide transformation, the main barriers for biomimetic solutions include material reproducibility, manufacturing cost, and standardization gaps. None of the entries in Table 1 show LCA (life cycle assessment) evidence, nor documented social impacts, indicating that environmental and societal validation trails behind technical proof-of-concept stages. Embedding these innovations into circular design frameworks and including policy instruments—such as targeted R&D tax incentives, green procurement, and technology demonstration funds—would accelerate readiness progression. The APC pathway’s reliance on innovation-driven carbon neutrality underscores the need for public private partnerships and mission-oriented programs to move these biomimetic systems beyond TRL 6.

(d) Readiness outlook for APC implementation

Given current TRLs, these technologies can be classified as medium readiness enablers—unlikely to deliver short-term emission reductions but capable of generating long-term transformative impacts by 2040–2050. Their incorporation into next-generation PV, thermal storage, and adaptive façade systems would strengthen APC alignment by introducing biologically optimized efficiency, self-regeneration, and multi-functionality—traits essential for the sustainable scaling of renewables.

Thus, while not yet deployed-ready, biomimetic energy technologies embody the innovation frontier required for the APC’s second half of the transition—bridging efficiency, resilience, and regenerative design. Strategic acceleration of validation, LCA integration, and demonstration pilots will determine their readiness to contribute meaningfully to a bio-inspiration integrated net zero energy system.

**Figure 5:**
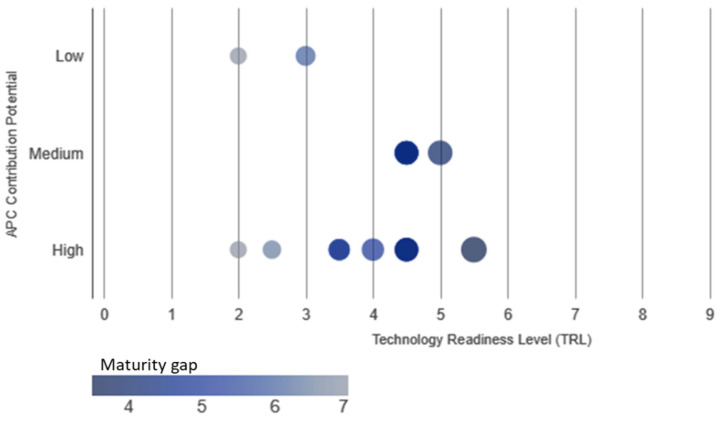
Readiness heat map: TRL vs. APC Contribution Potential of energy technologies in Table A3. X-axis (Readiness): Represents the Technology Readiness Level (TRL), indicating how mature the technology is (higher TRL means closer to commercialization). Y-axis (Potential): Represents the estimated APC Contribution Potential, categorized as Low, Medium, or High based on the domain’s impact on net zero efforts. Color (Heat/Maturity Gap): The color intensity corresponds to the Maturity Gap. Darker colors signify a larger gap, meaning the technology requires more developmental effort to reach full deployment (TRL 9).

Figure 5 shows the readiness heat map for the biomimetics technologies, presenting Technology Readiness Level (TRL) against APC Contribution Potential (an estimated proxy for impact), based on Table A3. The Maturity Gap is calculated as the difference between the maximum desired TRL (9) and the estimated TRL. A larger gap (darker color) indicates that the technology is further from commercial deployment.

Most energy generation technologies (high potential) fall within TRLs 3–5. This area is characterized by a high Maturity Gap, indicating that significant R&D is still needed to realize their full potential impact. Technologies in energy storage and water management (medium potential) generally have TRLs around 4–6. These are slightly more mature than the energy generation group, suggesting a comparatively smaller maturity gap and a shorter path to large-scale deployment. The low potential technologies are spread across the TRL range, including some with TRLs up to 6, such as microchannel heat sink, energy transport, energy management, material synthesis. This suggests a lower developmental risk but also a smaller assumed overall impact on the APC scenario.

## 7. Discussion

The proposed scenarios by the IEA, STEPS, and APC, clearly present today’s gaps to attain the net zero emissions target by 2050. The STEPS scenario shows the necessity of more rigorous policies and more commitment from the stakeholders to possibly approximate the APC scenario. Because of the intrinsic sustainability undertaken [1,5], biomimicry-based energy systems instead of biomimetic-based, which are more focused on the structures, present great promises regarding the technical aspects to develop full life cycle clean technologies and tackle most parts of the 2050 scenario needs: carbon capture, energy storage, and renewable energy generation [24], and optimize the energy management.

Hence, since the most bio-inspired approach employed in the literature for energy system design is the biomimetic solution-based approach, reviewing such designs and evaluating them under the biomimicry considerations may be the first step to bridging the gap for a transition to a full life cycle clean and sustainable system design. Moreover, biomimicry-based solutions’ features go deeper beyond sustainability, such as considering regeneration [5,37].

A second step could be implementing the problem-based approach proposed in [38]. Through such an approach, the circular economy features, and other full life cycle clean concepts can be integrated willingly into the final product or system. Such an example is presented in [39].

Finally, a third step needs to consider a way to measure the design’s success in being sustainable or even regenerative, e.g., the BiomiMETRIC assistance tool [40]. Yet, it seems that the 2050 scenarios give substantial care to the amount of carbon emissions above any other consideration, whereas a full life cycle carbon emission assessment may suffice for measuring the success of energy system designs.

Nevertheless, nature defines success as maintaining favorable conditions to sustain life over several generations by upcycling everything [1]. Thus, focusing only on reducing carbon emissions limits real success.

For this, the life cycle assessment could be of use [41]; however, such assessment only considers the product’s or system’s environmental impact, whereas sustainability also includes social welfare and economic harmonization [42].

In this context, a recent innovative approach gives insights into the potential relationship between bio-inspired solutions and energy transition systems (ETS) to assist the required full life cycle clean energy technologies for a sustainable energy transition. Such an approach frames this potential relationship through Common Boundary Concepts (CBCs) to categorize the theoretical foundations of said relationship [10].

These eight Common Boundary Concepts (CBCs) were adopted to link ecological and socio-technical perspectives in the bio-inspired energy transition framework (Table 2). These CBCs—ranging from socio-technical factors to land sink—provide a common vocabulary for interpreting how nature-inspired designs operate across niches, regimes, and landscapes. Integrating them clarifies the systemic position of each biomimetic solution and its potential contribution to a net zero energy transition. In addition, Table 1 provides proposed policy instruments with their applicability scale and purpose.

### 7.1. Policy Recommendations for Formalizing Bio-Inspired Integration in Strengthening a STEPS Pathway

Based on the synthesis that demonstrates that bio-inspired design is a necessary, though currently unstated, methodology for achieving NDC objectives, Table 2 presents recommendations for the next iteration of national climate commitments.

### 7.2. A Proposed Metric of Success for Bio-Inspired Energy Solutions

Table 3 identifies the essential cross-cutting indicators required to move beyond purely carbon-centric assessments and holistically compares bio-inspired energy solutions within the context of the APC pathway.

### 7.3. Proposed Roadmap

**Figure 6:**
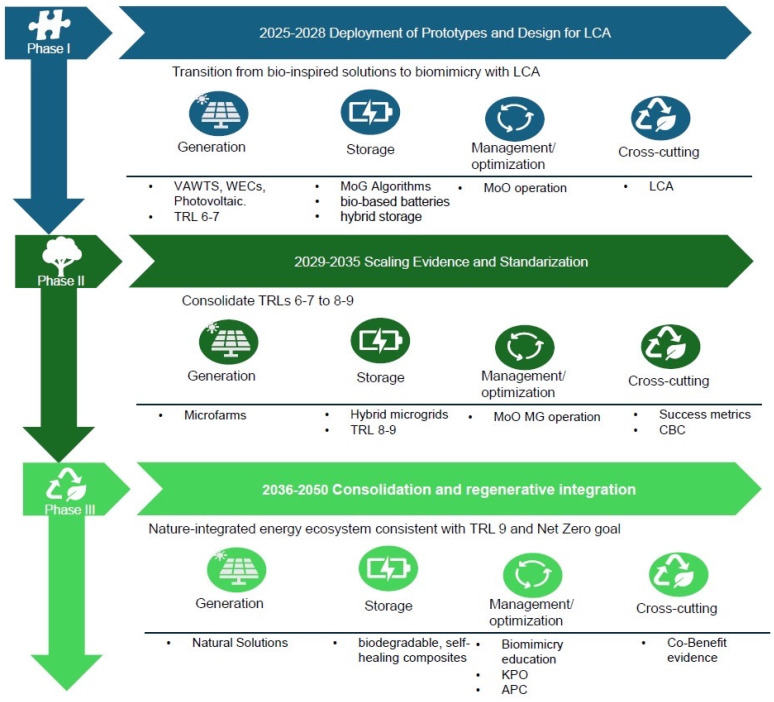
A proposed roadmap to show a possible progressive transition from bio-inspired approaches to systemic biomimicry, integrating life cycle assessment (LCA), ecological regeneration, and social value creation as core pillars of a nature-based energy transition toward net zero by 2050.

The proposed roadmap (Figure 6) illustrates a progressive transition from bio-inspired approaches to systemic biomimicry, integrating life cycle assessment (LCA), ecological regeneration, and social value creation as core pillars of a nature-based energy transition toward net zero by 2050.

It is structured into three temporal phases (2025–2050), each associated with increasing levels of technological maturity (TRLs 6–9) and four interlinked strategic domains: (i) energy generation, (ii) storage, (iii) management and optimization, and (iv) cross-cutting actions for sustainability, policy coherence, and circularity.

The roadmap’s purpose is to mandate a transition from this incremental development to a systemic, regenerative approach rooted in full life cycle biomimicry. This shift is achieved by simultaneously reforming governance and deploying new quantitative assessment tools.

(a) Phase I (2025–2028): Deployment of Prototypes and Design of LCA

This initial phase marks the transition from bio-inspired to biomimetic design, where early prototypes integrate environmental performance through LCA metrics and eco-design principles.

At the generation level, prototypes of vertical axis wind turbines (VAWTs), wave energy converters (WECs), and photosynthesis-inspired photovoltaic cells operate at TRLs 6–7, demonstrating aerodynamic and photonic advantages derived from natural morphologies. In energy storage, multi-objective genetic (MoG) algorithms are introduced to optimize bio-based batteries and hybrid storage systems inspired by lignin and cellular structures. In management and operation, multi-objective operation (MoO) strategies emulate natural cooperation networks (e.g., swarm or mycelial intelligence) to enhance energy distribution and self-optimization. Cross-cutting efforts emphasize circular design, life cycle transparency, and the establishment of bio-informed decision frameworks linking engineering and environmental policy.

Outcome: Transition from conceptual inspiration to validated biomimetic prototypes with quantifiable environmental performance and circularity potential.

(b) Phase II (2029–2035): Scaling, Evidence, and Standardization

The second phase consolidates biomimetic approaches through technological upscaling, hybridization, and standardization in sectors with demonstrated value and societal acceptance.

Generation systems advance through modular micro-farms combining wind, solar, and ocean energy using biomimetic surfaces (e.g., lotus effect coatings, coral-inspired geometries). Storage solutions evolve into hybrid microgrids incorporating hierarchical architectures inspired by vascular or mycelial systems, reaching TRLs 8–9. Operational frameworks (MoO MG) enable distributed optimization and adaptive control, enhancing resilience and reducing losses through bio-inspired coordination algorithms. The cross-cutting dimension integrates success metrics and community co-benefit (CBC) models, linking technological performance with social well-being and ecosystem restoration.

Outcome: Establishment of standardized protocols, performance indicators, and policy readiness for biomimetic technologies, fostering credibility and scalability within global energy systems.

(c) Phase III (2036–2050): Consolidation and Regenerative Integration

The final phase envisions the full adoption of biomimicry as a regenerative paradigm, merging technological innovation with ecological restoration and inclusive governance.

Natural solutions for generation, such as bio-integrated solar façades, wave reef hybrid systems, or mangrove-inspired coastal infrastructures, coexist with performance-based markets that reward low-impact, high-efficiency designs. Energy storage and materials systems incorporate biodegradable, self-healing composites and metabolic energy cycles that mimic photosynthesis and nutrient loops. At the management level, biomimicry education, knowledge performance outcomes (KPOs), and adaptive governance frameworks (APC) promote resilience and continuous learning. Cross-cutting actions focus on co-benefit verification, aligning energy production with biodiversity recovery, circular economy metrics, and social empowerment.

Outcome: Maturity of a regenerative, nature-integrated energy ecosystem (TRL 9) that supports net zero goals, balances human development and ecological integrity, and operationalizes biomimicry as governance.

Across the three phases, the roadmap defines a scalable trajectory where technological innovation, ecological circularity, and social value evolve in parallel. The integration of biomimicry, nature-based solutions (NBS), and LCA-driven circularity fosters a systemic model for sustainable development.

### 7.4. Main Limitations

This work presents a compelling framework, but as a perspective proposing a novel policy and assessment shift, its primary limitations lie in the scope of evidence and the practical implementation details.

(a) Limited Empirical Validation (TRL Gap):
  - The core argument relies on the low TRLs (3–6) of biomimetic solutions. However, the work does not include a detailed, longitudinal analysis of successful scaling efforts to determine the specific, systemic obstacles (beyond general “policy gaps”) that have historically prevented these technologies from moving from TRL 6 to TRL 9. No patent or utility models were looked for at this stage in the research due to difficulties in assessing viable functionality and lack of theoretical description and evaluation evidence.
  - The paper establishes the Maturity Gap but does not present a case study demonstrating how the proposed FPI or LCSI metrics would have accelerated a technology’s passage through the phase between R&D funding and commercialization.
(b) Assessment of Economic and Social Integration:
  - While the paper proposes the LCOE-B and SRC Avoided metrics, the economic models for quantifying the long-term, distributed benefits of resilience and self-healing (core biomimicry advantages) are not provided. These benefits are complex to monetize and are often seen as “externalities” by traditional investors.
  - The social aspect mentioned in the introduction, is primarily addressed by the CBCs (Common Boundary Concepts) but lacks specific socio-economic indicators that can measure community acceptance, equitable access, or job creation specific to the biomimetic supply chain.
(c) Policy Implementation Specificity:

The call to integrate NbI (Nature-based Innovation) into NDCs is ambitious. The work does not detail a practical, phased policy roadmap, for example, which specific NDC sections need amending, or what global governance mechanism (e.g., IPCC or UNFCCC working groups) should pilot the adoption of the LCSI metric.

### 7.5. Future Directions

The limitations of the current work naturally point toward avenues for future research that can solidify the paper’s framework and accelerate its policy impact.

(a) Develop Standardized Quantitative Tools:
  - Prioritize the development and calibration of the Life Cycle Sustainability Index (LCSI) and the Functional Performance Index (FPI). Future work should create open-source, user-friendly toolkits (e.g., a BiomiMETRIC software tool) that allow researchers and policymakers to calculate these metrics consistently, enabling true cross-technology comparison.
  - Develop standardized economic models to quantify the long-term monetary benefits of SRC Avoided and MIR in large-scale energy infrastructure projects, making a clear business case for biomimicry.
(b) Longitudinal Case Studies and Pilot Programs:
  - Conduct in-depth longitudinal case studies on biomimetic technologies that have successfully passed TRL 7. Analyze the policy and financial mechanisms that did or could have facilitated their scaling, using the proposed FPI, MIR, and LCSI metrics in a retrospective analysis.
  - Propose and execute pilot demonstration projects in collaboration with national or regional energy agencies to test the efficacy of the NbI policy classification and the new metrics (LCSI) in attracting targeted Green Bond financing.
(c) Refine Policy and Governance Roadmap:
  - Focus on generating explicit policy language for integrating biomimicry into global governance frameworks. This includes drafting model text for NDCs or national energy transition acts that explicitly mandate reporting on FPI and MIR for publicly funded energy projects.
  - Further elaborate on the Common Boundary Concepts (CBCs) to develop a framework for stakeholder engagement that ensures the regenerative principles of biomimicry translate into tangible, positive social and ethical outcomes.

## 8. Conclusions

The divergence between the conservative Stated Policies Scenario (STEPS) and the ambitious Announced Pledges Case (APC) clearly defines the required pace and scope of energy innovation. To close this critical gap and realize net zero targets, incremental technological change is fundamentally insufficient; instead, a systemic, nature-based paradigm shift is required. This perspective paper establishes that biomimicry is the methodological proxy for this acceleration, offering a framework that moves beyond the limitations of current carbon-centric policy and assessment tools.

To align with the APC successfully, the energy sector must commit to moving beyond solely developing structure-focused biomimetic systems and fully embrace sustainability- and regeneration-focused biomimicry-based energy systems. This transition is paramount because true biomimicry inherently addresses the technical requirements for full life cycle clean technologies—encompassing generation, storage, and management—by ensuring solutions are sustainable and regenerative. Furthermore, this approach addresses the critical measurement gap by adopting nature’s standard of success (sustaining life over generations) rather than relying solely on minimizing single metrics, which often fail to capture broader societal and environmental impacts.

To institutionalize this rigor and de-risk the necessary investment to push bio-inspired innovations beyond TRL 6, governance must be purposefully adapted. This involves reframing policy by transitioning from the limited Nature-based Solutions (NbS) to Nature-based Innovation (NbI) into key instruments like Green Bonds and Technology Needs Assessments (TNAs), thereby mandating the inclusion of biomimicry principles. Crucially, this requires quantifying regeneration through the introduction of novel metrics—specifically the Material Intensity Reduction (MIR), the Functional Performance Index (FPI), and the Life Cycle Sustainability Index (LCSI)—which provide the essential “metrics of success” yardstick for measuring resource parsimony, resilience, and regenerative capacity.

Ultimately, by strategically accelerating the validation process and formalizing Biomimicry principles into policy, these currently low-TRL technologies can transition from medium-readiness enablers to long-term transformative systems. The final step of systemic integration, guided by Common Boundary Concepts (CBCs), ensures that these innovations are contextually sound and contribute holistically to community well-being and ecological health. This comprehensive strategy fulfills the innovation frontier required for the APC’s second half of the energy transition, securing a truly regenerative and resilient path toward net zero.

## Figures and Tables

**Table 1:** Integration of common boundary concepts (CBCS) with biomimicry principles and policy instruments for a nature-positive energy transition.

CBC [10]	Proposed Function of the Coupled Bio-Inspred and ETS [10]	Biomimicry Principle/Pinnacle Analogy	Interpretation for Energy Transition	Policy Instrument	Instrument Applicability Scale **	Instrument Purpose/Function ***
Socio-technical factors	Respond to global societal and environmental challenges such as land rush, uneven distribution, and the lack of budget as cost-benefit solutions.	Symbiotic cooperation among species ensuring shared benefits within limited ecosystems.	Bio-inspired governance mechanisms emulate mutualism and resource sharing, enabling fair access to clean energy.	Global carbon tax *, international climate finance, inclusive energy governance frameworks.	Global/supranational	Establish shared accountability and equity in emissions reduction; ensure fair resource distribution and financial support for developing nations.
Urban infrastructure regime	Help stakeholders and landowners to use aggregated incentives and other available financial and institutional re-sources.	Rewarding species that enhance system-wide efficiency (e.g., mycorrhizal networks).	Biomimetic coordination in smart grids and distributed networks modeled on cooperative biological systems.	Green infrastructure bonds, public–private partnerships, adaptive regulatory frameworks.	National/metropolitan	Mobilize capital and foster institutional collaboration for adaptive, bio-inspired energy and infrastructure systems.
Social innovation (niche)	Coupled solar panels and green infrastructure may have multiple benefits in terms of better cooling effects and less environmental disturbances.	Mycorrhizal fungi and underground networks distribute nutrients and information for mutual resilience.	Represents local biomimicry niches that incubate transition pathways at pilot scales.	Living labs, innovation vouchers, community energy cooperatives.	Local/regional	Promote participatory experimentation and co-creation of bio-inspired energy solutions; accelerate niche innovation and social learning.
Natural capital	People can become familiar with both renewable energy technologies and natural infrastructure living labs at the same place and access real time data.	Regeneration and care of progeny in ecosystems; principles of strong biomimicry.	Regenerative design replenishes ecosystems while producing energy.	Ecosystem service valuation, biodiversity credits, payments for ecosystem services (PES).	Regional/national	Internalize ecological value within energy and economic planning; incentivize regenerative and restorative design.
Energy landscape	Smoothly transform traditional infrastructure to new natural infrastructure, e.g., use contaminated lands as natural capital for renewable energies.	Tree leaves and soil organisms recycle carbon and nutrients.	Bio-inspired land use that transforms energy landscapes into regenerative carbon sinks.	Land use planning reforms, renewable zoning, restorative energy programs.	Regional/municipal	Integrate bio-inspired land management and renewable infrastructure, transforming degraded areas into regenerative energy landscapes.
Resource management	Support both waste and resources management by using less land, efficiency, or using more sustainable resources.	Mycelium networks and circular metabolism in ecosystems.	Circular energy loops emulating natural metabolism for waste minimization and nutrient reuse.	Circular economy regulation, industrial symbiosis platforms, eco-labeling standards.	National/sectoral	Promote closed-loop material and energy flows across industries, enhancing efficiency and reducing waste.
Urban ecosystem services	Support more services in terms of ecosystem restoration and conservation, energy efficiency and learning services in urban areas for people.	Cooperative adaptation and interspecies resilience in ecosystems.	Bio-inspired energy infrastructures deliver co-benefits (cooling, air quality, biodiversity).	NbS design guidelines, urban restoration funds, environmental education policies.	Municipal/community	Enhance biodiversity, microclimate regulation, and social well-being through nature-based urban energy interventions.
Land sink	Reduce embodied carbon emissions by using natural environments, land, and renewable energies.	Soil organisms and carbon-sequestering plants converting waste into resources.	Integrates biomimicry for carbon capture and regenerative land-energy systems.	Carbon pricing *, soil carbon credits, regenerative agriculture incentives.	National/local (rural)	Stimulate carbon sequestration and restoration practices that transform land into active carbon sinks supporting net zero goals.

CBC: shared conceptual “boundaries” linking NbS and energy transitions; Regime: dominant practices/technologies and rules; Niche: protected spaces where novelties mature. * “Carbon pricing” is used as a general term covering carbon taxes and emissions trading schemes at national or sectoral levels. The “global carbon tax” refers specifically to a coordinated international pricing mechanism addressing socio-technical inequities in the net zero transition. ** Scale of applicability helps readers see who implements each instrument, from the UN or International Monetary Fund (IMF) (global) down to municipalities or communities (local). *** Purpose reveals why each instrument matters and its function within a regenerative, bio-inspired transition pathway.

**Table 2:** Proposed recommendations to integrate bio-inspiration into policies.

Challenges	Recommendation/Action Area	Core Requirement/Mechanism	Rationale/NDC Goal Alignment
Formalizing Bio-Inspired Design Language in NDCs			
Without formal recognition of biomimetics, there is a risk that countries will meet their NbS quotas with minimal, easily implemented projects, failing to drive the deep technological and systemic shifts required for long-term climate resilience.	Elevating NbS to NbI (Nature-based Innovation)	Explicitly recognize Nature-based Solutions (NbS) as the foundation for Nature-based Innovation (NbI), encouraging the systematic extraction of functional, resource-efficient principles via biomimetics.	Compels rigorous R&D and interdisciplinary planning, ensuring NbS is not relegated to minimal projects.
NDC implementation requires “new and innovative approaches to unlock finance and support for developing country Parties at scale” and relies heavily on technology transfer [29]. Investors are often hesitant regarding complex, novel climate technologies.	Explicit Technology Transfer Pathways	Mandate the inclusion of specific biomimetic and bionic research and development (R&D) in Technology Needs Assessments (TNAs).	Elevates technologies, such as AI-driven bionics for lightweight material optimization [43], to nationally supported climate technologies eligible for international finance and transfer.
Increases ambition beyond the current low 28% CE explicit mention rate [44].	Specific Circularity and Regenerative Targets	Introduce mandatory quantitative targets for resource efficiency based on biological standards; promote bio-based materials and mandate Life Cycle Assessment (LCA) tools.	Ensures evaluation of material choices in infrastructure projects via indices comparable to natural systems.
Financing the Bio-Shift and Measuring Impact			
Strive for bio-inspired design-dedicated financing mechanisms and rigorous, tailored performance metrics, such as the ones proposed in [45,46].	Blending Finance for Bio-Innovation	Commit to leveraging national budgets, private investment, and multilateral funds (GCF) to de-risk pilot projects, e.g., bio-adaptive infrastructure [47] (bio-adaptive coastal protection zones or self-regulating, and passively cooled urban structures [48]).	Unlocks new finance and support via mechanisms like Green Bonds earmarked for innovation demonstrating biomimetic/bionic resource efficiency gains.
	Developing Mitigation Metrics	Integrate Key Performance Indicators (KPIs): tracking Material Intensity Reduction (MIR) and Embodied Carbon Efficiency (ECE) achieved through bionic optimization [43].	Provides a quantifiable link between bio-inspired structural efficiency and industry/building sector mitigation goals.
	Developing Adaptation Metrics	Integrate Key Performance Indicators (KPIs): measure resilience using a Functional Performance Index (FPI) assessing structural longevity, self-repair, and multi-functional ecological benefits.	Provides a robust comparison against conventional infrastructure.
Integrating Biomimicry into Capacity Building			
Many parties have identified capacity needs specifically related to technology deployment. Energy demand or urban metabolism reduction via more “educated” users.	Curriculum Standardization (ACE)	Integrate biomimicry and ecological literacy as required components of national Climate Action Education (ACE) strategies [49,50,51].	Directly addresses the need for systems thinking and knowledge transfer, empowering youth as future drivers of the green economy and net zero commitments.
The development of the specialized workforce needed for the green economy depends on incorporating bio-inspired systems thinking into capacity building efforts.	Targeted Professional Training	Establish standardized training programs for architects, engineers, urban planners, and manufacturers focused on bio-based materials, LCA, passive biomimetic cooling, among others.	Ensures the workforce is equipped to implement regenerative design at scale [34].

**Table 3:** Proposed indicators per criterion to evaluate the performance of bio-inspired energy solutions.

Criterion	Indicator	Unit/Assessment	Relevance to Biomimicry/APC	Justification	Reference Context
Technical	Functional Performance Index (FPI)	Dimensionless index or specific output (e.g., kWh/kg)	Quantifies the multi-functionality (design excellence) and efficiency relative to a conventional baseline.	Quantifies the success of emulating nature’s multi-functionality and performance-per-mass, which is a core tenet of efficient biological design. Critical for lightweight, integrated energy systems.	Biological Multi-Functionality: The ability of a single structure like a leaf to optimize fluid flow, gas exchange, and energy harvesting simultaneously.
	Material Intensity Reduction (MIR)	Percentage reduction	Tracks the reduction in virgin material mass required per unit of function, focusing on resource efficiency and lightweighting.	Directly addresses the resource demands of the energy transition. Biomimicry often uses scarce, locally available materials, enforcing resource efficiency and reducing embodied carbon (e.g., using hierarchical structures to achieve strength with minimal mass).	Nature’s Material Economy: The efficient use of common elements like carbon, hydrogen, and oxygen over scarce metals. Eco-efficiency metrics.
Environmental	Life Cycle Sustainability Index (LCSI)	Index (0–1) based on ISO 14040 and ISO 14044	Measures the regenerative potential by assessing full cradle-to-cradle impact, including biodegradability and toxicity, moving beyond CO_2_ equivalence.	Expands traditional LCA to measure the regenerative potential by including metrics on circularity, toxicity, and end-of-life biodegradability, aligning with the ‘cradle-to-cradle’ ethos of true biomimicry.	Cradle-to-Cradle Design: The framework of William McDonough and Michael Braungart, requiring products to be either biological or technical nutrients.
	Water Footprint Reduction (WFR)	Liters of water saved per unit of energy	Critical for resilience and sustainable operation in water-scarce regions, inspired by nature’s water management.	Water scarcity is a critical resilience factor for future energy systems (e.g., cooling power plants). Emulating nature’s closed-loop water strategies (e.g., fog harvesting) ensures the energy solution is ecologically compatible.	Nexus Thinking: The energy-water-food nexus where reducing water use improves system resilience. Biological water management (e.g., desert beetles).
Economic	Levelized Cost of Energy (LCOE-B)	EUR€/MWh or USD$/MWh	LCOE adjusted for biomimetic benefits (e.g., lower maintenance and materials costs) over the system’s full lifetime.	A standard economic metric, but adjusted to factor in the long-term cost benefits of biomimicry, such as reduced maintenance costs (due to self-cleaning/healing) and lower material input (MIR).	Full Cost Accounting (FCA): Moving beyond initial capital expenditure to include externalities and lifetime operational savings.
	System Resilience Cost (SRC) Avoided	Monetary value of avoided losses/damages	Quantifies the economic value of increased reliability due to biomimetic design (e.g., anti-fouling, self-healing).	Translates the reliability and robustness benefits into a financial value. A key metric for de-risking investment in high-impact, novel solutions needed for the APC.	Value of Lost Load (VoLL): A metric used in grid planning to quantify the economic impact of power outages.
Reliability/Robustness	Technology Readiness Level (TRL)	TRLs (1–9)	The standard measure of maturity required to guide technology choices and de-risk investment for the APC acceleration.	The essential criterion for guiding policy and funding choices. It identifies the necessary developmental effort (Maturity Gap) required to meet the rapid deployment goals of the APC.	NASA/DoD Standard TRL Scale: A universal tool for comparing technological maturity and risk.
	Self-Healing/Adaptability Score (SHAS)	Index or MTTR reduction (%)	Assesses the system’s inherent ability to recover from damage (self-healing) or adapt to changing conditions (e.g., flow, temperature), a core tenet of biological systems.	Directly assesses the biological imperative for survival and resilience through autonomous repair and flexible response to environmental change. Essential for minimizing downtime in critical energy infrastructure.	Nature’s Strategies for Resilience: Autonomic repair mechanisms (self-healing polymers) and optimal network topologies (Physarum polycephalum).

**Figure A2:**
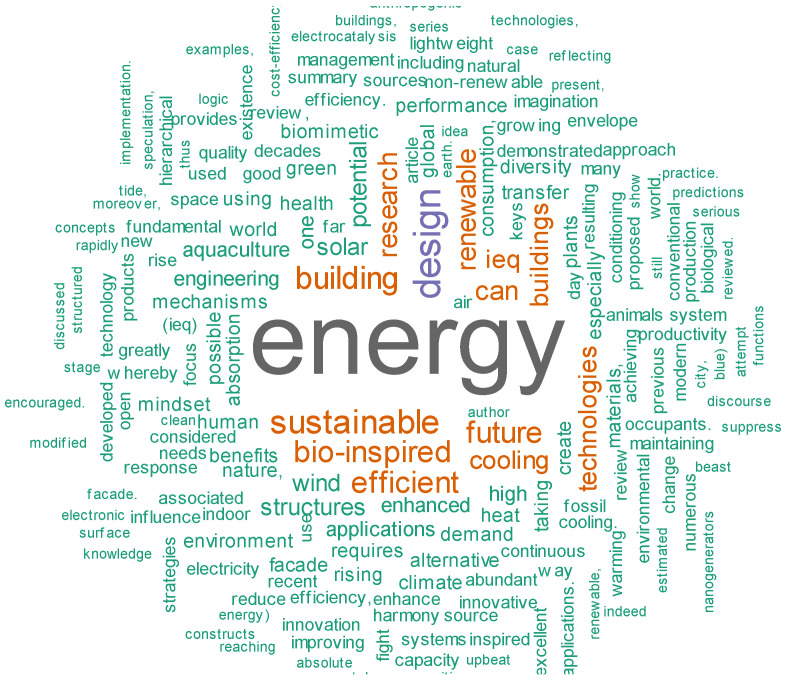
Word cloud of the motivation of various research documents.

**Figure A3:**
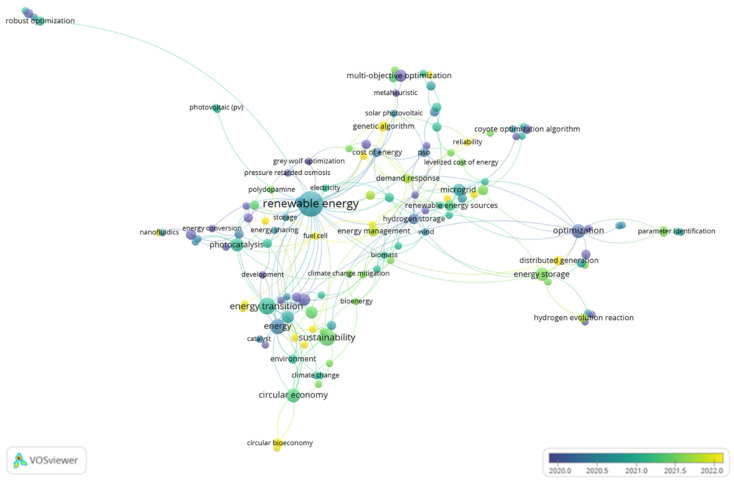
Overlay visualization of keywords in research documents regarding energy systems and bio-inspiration. This keyword map elaborated in VOSviewer (version 1.6.20) presents the temporal trending of the thematics, from old-used terms (purple colored) to newest-used terms (yellow colored).

## Data Availability

The original contributions presented in this study are included in the article/Supplementary Materials. Further inquiries can be directed to the corresponding author.

## Supplementary Materials

The following supporting information can be downloaded at https://www.mdpi.com/article/10.3390/biomimetics10120842/s1, Table S1: Summary of the approaches, pinnacles and objectives by energy system.

## Nomenclature

Announced Pledges Case	APC
Carbon capture, usage, and storage	CCUS
Circular economy	CE
Common Boundary Concepts	CBC
Energy transition systems	ETS
Global Industry Classification Standard	GICS
Greenhouse gas	GHG
International Energy Agency	IEA
Life cycle assessment	LCA
Nature-based solutions	NBS
Net zero emissions	NZE
Nationally Determined Contributions	NDC
Plant-Microbial Fuel Cell	PMFC
Stated Policies Scenario	STEPS

## Appendix A

This section allocates the evidence found in the current literature employing either biomimetics or biomimicry in their system design for energy generation and energy storage, or for energy management and optimization.

Moreover, the literature searching protocol is presented as well as a codebook to better understand the classifications of the Technology Readiness Level (TRL) and bio-inspired concepts and terminology.

### Appendix A.1. Operational Codebook

To ensure consistent interpretation, each example was tagged using an operational codebook defining both the evidence level (validation stage and TRL, as in Table A1) and the type of nature-based design approach (biomimetics, biomimicry, weak biomimicry, as in Table A2 and Figure A1). The codebook allows reproducibility and comparability across Table A3 and Supplementary Information.

**Table A1:** Technology Readiness Level (TRL) categories [52,53].

TRL	Definition	Typical Validation Level
1	Basic principles observed	Theoretical/concept only
2	Technology concept formulated	Analytical/early model
3	Experimental proof of concept	Lab-scale, basic validation
4	Technology validated in lab	Controlled lab testing
5	Technology validated in relevant environment	Lab + partial system integration
6	Prototype demonstrated in relevant environment	Small pilot/integrated subsystem
7	System prototype demonstration in operational environment	Field pilot
8	System complete and qualified	Real-world validation complete
9	System proven in operational use	Commercial deployment

**Table A2:** Proposed codebook for bio-inspired design concepts and terminology [5,46,54].

Category *	Working Definition	Tagging Rule
Nature-inspired	Broad category where nature serves as a conceptual or aesthetic inspiration rather than a direct biological model. Does not require scientific analogy or biological fidelity.	Tag when nature influences general orientation, shape, or concept, but no specific organism/process is clearly emulated.
Nature-based solutions (NbS)	Actions that use (or restore) natural ecosystems to address societal challenges (climate mitigation, resilience, biodiversity, sustainable infrastructure). Based on ecosystem use, conservation, or restoration.	Tag when the intervention uses real ecosystems, vegetation, soil, water cycles, ecological restoration, or hybrid green–grey infrastructures. This is not biomimicry but a complementary category.
Bio-inspired	Uses biological principles as conceptual heuristics to guide engineering design, often at the abstract level (patterns, networks, distributed intelligence).	Tag when the analogy is conceptual, mathematical, or systemic (e.g., “ant colony optimization algorithm,” “mycelium-like networks”).
Bionics	Applies biological functions to technical systems with a strong engineering and cybernetic lens. Often mechanistic and focused on translating biological performance into devices/sensors.	Tag when biological functions are converted into mechanical, robotic, or electromechanical systems (e.g., artificial muscles, insect-wing-inspired micro-robots).
Biomimicry (regenerative/life cycle)	Umbrella category covering both weak and strong biomimicry. Focuses on using nature’s principles to restore, sustain, or enhance material, energy, or ecological flows.	Use when the design logic follows ecosystem behavior rather than isolated structural mimicry.
Biomimetics (form/function)	Engineering approach that imitates natural structures, mechanics, or geometries to improve performance, efficiency, or durability. Does not integrate system-level ecological principles.	Tag when the solution copies a form, pattern, or mechanism from nature to optimize a technical function (drag reduction, antireflection, cooling, etc.).
Weak biomimicry	Uses biological ideas or metaphors superficially, without achieving measurable ecological, regenerative, or systemic benefits.	Tag when sustainability effects are unverified, incidental, or symbolic, even if nature is referenced.
Strong biomimicry	Emulates natural processes, ecosystem dynamics, or life cycles to produce regenerative, closed-loop, or self-sustaining outcomes.	Tag when the solution shows measurable life cycle benefits, restorative effects, circularity, or ecosystem integration.
Pinnacle (biological analog)	The specific organism, biological structure, ecosystem, or natural process that inspires the design. Represents the natural reference model.	Always specify the pinnacle in the case description (e.g., “lotus leaf → self-cleaning PV coating”).

* Categories are hierarchized in Figure A1.

### Appendix A.2. Literature Searching Protocol

We queried Google Scholar and ScienceDirect databases for 2010–2024 with strings combining energy-function terms and bio-inspiration terms, e.g., TITLE-ABS-KEY((biomimic* OR bio-inspire* OR bionic*) AND (photovoltaic OR wind OR wave OR battery OR storage OR microgrid OR optimization)). More details are presented as follows:

- Databases: Google Scholar and ScienceDirect.
- Time window restriction: 2010–2025.
- Keyword combination query: (energy generation OR energy storage OR energy management OR optimization) AND inspired AND (nature OR biomimicry OR biomimetic) NOT architecture
- Screening: Title/abstract, then full text.
- Types of documents accepted: Gray literature and peer-reviewed documents such as books, book chapters, scientific articles, thesis, and conference papers.
- Inclusion criteria: Studies with at least one level of system validation such as simulation (not just the idea), English language, peer-reviewed, energy-relevant (generation/storage/management), and reports a mechanism, metric, or validation.
- Exclusion criteria: Non-energy applications (studies related to architecture, biophilic considerations), studies with purely metaphorical uses without mechanism, and review articles unless they contain primary metrics.
- Extraction fields: Domain, pinnacle, approach (biomimetics/biomimicry), device/system, objective metric, validation level, maturity, LCA evidence, social impact evidence, and citation.

This led to a total of 30 relevant peer-reviewed papers: Energy generation (N = 19), Energy storage (N = 7), and Energy management (N = 4). Finally, no patent or utility models were looked for at this stage due to difficulties in assessing viable functionality and lack of theoretical description and evaluation evidence.

### Appendix A.3. Current Evidence on Bio-Inspired System Design for Energy Generation, Energy Storage and Energy Management

#### Appendix A.3.1. Key Aspects When Considering How Nature Acts in the Design of Energy Systems

The literature indicates that the main reason researchers look at nature for inspiration is to develop more sustainable, cleaner, environment-friendly ways of harvesting energy.

Figure A2 shows an extract of most occurrence terms found in the motivation of various research documents analyzed here. The terms “energy” and “design” are highlighted among all terms, but the terms “bio-inspired”, “sustainable”, “efficient” and “future” appear with similar frequency, helping realize researchers’ reasons to look for inspiration in nature. Moreover, renewable technologies are of interest, where the two most common energy harvesting sources appear to be “solar” and “wind”. This agrees with [24]. The appearance of the term “building” as frequently as “sustainable” suggests an increased interest in energy systems in building-level strategies such as distributed energy generation.

In fact, such interest in distributed generation along with bio-inspiration, considering the circular economy, is currently trending (yellow color in Figure A3). The latter arises because of the frequency of the appearance of the terms “bio-inspired” (in orange color) and “biomimetic” (in light green color), which may appear similar, but the fundamental aspects of the former, which concentrates on an idea to be extracted, are part of the latter, which strives to directly imitate nature, and the way a species works to an application [5]. This leads to a need for a comprehensive review of such research documents to clarify such a presumption. Table A3 showcases a summary of the biological analogies (pinnacles) used as inspiration for the objective or improvement, classified by the bio-inspired approach and energy system found in the literature analyzed.

**Table A3:** Summary of the approaches, pinnacles and objectives by energy system. Refer to Supplementary Documentation, Table S1, for complete information about each energy system reviewed.

Domain	Pinnacle	Approach	Energy System/Device	Validation Level	Maturity	Ref.
Energy generation	Central fovea (retina)	Biomimetics	Thin-film PV/light-trapping textures	Simulation/Lab	Concept–Prototype	[55]
Energy generation	Coleoptera beetle scales	Biomimetics	Thin-film PV/light-trapping textures	Simulation/Lab	Concept–Prototype	[55]
Energy generation	Pink butterfly wings	Biomimetics	Thin-film PV/light-trapping textures	Simulation/Lab	Concept–Prototype	[55]
Energy generation	Moth eye nanostructure	Biomimetics	PV cover/AR surface	Lab	Prototype	[56]
Energy generation	Lotus leaves, rice leaves and water strider legs (superhydrophobic)	Biomimetics	PV self-cleaning film	Lab	Prototype	[8]
Energy generation	‘Sweat’ cooling strategy	Biomimicry	PV panel cooling gel	Lab/Small pilot	Prototype	[7]
Energy generation	Leaf anatomy/photosynthesis	Biomimicry	Leaf-mimic PV light-capturing layer	Lab	Prototype	[57]
Energy generation	Eastern hornet queen	Biomimetics	Electrical cell	Lab	Basic research	[58]
Energy generation	Protein nanowires (Geobacter)	Biomimicry	Ambient humidity harvester	Lab	Concept–Prototype	[59]
Energy generation	Bacteria	Biomimetics	Microbial Fuel Cell	Lab	Prototype	[60]
Energy generation	Bird feathers (movement)	Biomimetics	Kinetic façade energy harvester	Simulation	Concept	[61,62]
Energy generation	Owl feathers and the tubercles of humpback whale fins	Biomimetics	Solves aerodynamic noise	Lab testing	Laboratory component prototype.	[61]
Energy generation	Nelumbo nucifera (Sacred Lotus)	Biomimetics	Horizontal-axis wind blade	Simulation + Experiment	Prototype	[63]
Energy generation	Maple seed leaf	Biomimetics	Blades in vertical axis turbines	Simulations	Concept/Simulation	[64]
Energy generation	Fish schooling	Biomimetics	Vertical axis wind turbines array layout	Simulation/Theory	Concept	[65]
Energy generation	Rock crab legs	Biomimicry	Wave power turbine-generator systems	Concept/prototype	Concept	[66]
Energy generation	Sea fauna mix	Biomimetics	TALOS device	Theoretical application	Concept–Prototype	[67]
Energy generation	Leaf flutters (Palm leaf)	Biomimicry	Wind energy collectors (Artificial blades)	Wind tunnel tests	Prototype	[68]
Energy generation	Metamaterials (Defective state)	Biomimetics	Ocean monitoring systems (Wave energy harvester)	Simulation/Lab	Prototype	[69]
Energy storage	Photosynthesis (system-level emulation)	Biomimetics	PV + electrochem. H2 modules	Lab system integration	Concept–Prototype	[70]
Energy storage	Wood hierarchical porosity	Biomimetics	Battery components (separators etc.)	Lab	Prototype	[71]
Energy storage	Lignin quinones; Venus flytrap cage	Biomimetics	Graphene-reconfigured lignin cathode	Lab	Prototype	[72]
Energy storage	Sucrose-derived carbon	Biomimetics	Li4Ti5O12 anode modification	Lab	Prototype	[73]
Energy storage	Polysaccharides/ingestible design	Biomimetics	Edible battery + nanogenerator	Lab	Prototype	[74]
Energy storage	Plant leaf veins (Fractal dimensions)	Biomimetics	LHTES systems (Vertically aligned annular fins)	Numerical and experimental validations	Small pilot/Prototype	[75]
Energy storage	Conch shell (Spiral fins)	Biomimetics	Phase change capsule (Thermal energy storage)	Simulation	Concept–Prototype	[76]
Energy management	Ant pheromone foraging	Biomimicry	Dispatch/routing algorithms	Simulation	Concept	[77]
Energy management	Mold physarum network growth	Biomimicry	Network design/microgrids	Lab analog/Simulation	Concept	[77]
Energy management	Mold physarum network growth	Biomimicry	Transportation organization	Laboratory experiments with living physarum polycephalum plasmodium using physical analogs (oat flakes representing cities).	Analog lab model/Proof-of-Concept.	[78,79]
Energy management	Particle swarm optimization (PSO)/Genetic algorithm (GA)	Biomimetics	Renewable energy system optimization (University microgrid)	Theoretical application/Simulation	Concept	[80]

#### Appendix A.3.2. Energy Generation

Most advancements in energy generation systems focus on replacing the existing technology rather than improving it via thin films [55], self-cleaning [81], and self-cooling [7]; still, a few focus on new patterns to optimize existing technologies while maintaining the use of the known power sources for energy generation, i.e., solar, wind, hydro, waves, and biomass. The most exploited natural energy source is solar, followed by wind, and tidal power, all of which are conditioned by solar effects. Improvements to the current photovoltaic systems, developing thinner film cells, are found to be inspired by the central fovea in the retina of the human eye, the natural photonic structure with fluorescent molecules embedded within the multilayer structure of the scales of the beetle *Hoplia coerulea* (Coleoptera), and the microscopic structure of the wings of the pink butterfly (*Pachliopta aristolochiae*) [55]. The development of less reflective surfaces constitutes another improvement to capture more light inspired by the principles in moth eyes [56]. Also, the use of superhydrophobic surfaces inspired to the lotus leaves was demonstrated to also be effective for keeping the photovoltaic panel surfaces unstained and therefore increase their efficiency [8].

Finally, since solar panels only need sunlight to produce electricity and provide optimal conditions under a clear sky at a temperature of 25 °C, excessive heat reduces their efficiency: this obviously affects panels in hot climates more than in cold climates. This disadvantage could be solved with a gel that allows the solar cells to “sweat” by increasing convective heat exchange with the surrounding air cooling the panels, and helping to increase power generation by up to 19% [7].

The advancements concerning solar power focus on new developments to enhance the converting and capturing of solar energy while intending to build cleaner technologies than conventional photovoltaic solar panels. This has led researchers to a deep understanding of one most known solar harvesting processes, the photosynthesis process in plants [57,82]. Using chemical reactions, structure, and behavior, plants maximize the harvesting and converting of solar power, yet the chemical reaction behind photosynthesis is a slow process. Significant improvements are achieved by mimicking plant leaves’ structure and anatomy, creating a light-capturing layer along with an epidermis-like layer, increasing efficiency by up to 55% compared to conventional [57].

Other organisms also present similar characteristics to generate electricity. The features of the silk of nests from the eastern, or oriental, hornet (*Vespa orientalis*) queen helped develop an electrical cell capable of generating and storing electricity. Moreover, although not yet entirely understood, the hornet itself can produce and store electricity through its brown and yellow pigments [58,83].

New developments are found in fewer studies generating energy from other sources, e.g., humidity in the air [59] and wastewater [60]. Bacteria can serve as catalysts to convert chemical energy into organic matter and then into electrical energy [60,84] when, for instance, implemented in developed microbial fuel cells, which convert energy from wastewater [60]; however, low energy output limits applications. The most studied microorganisms for these aspects are *Geobacter sulfurreducens* [59] and *Shewanella oneidensis* [84]. A combination of plants and bacteria can enhance energy generation [82] when the plant waste is used by bacteria, as mentioned earlier, thus increasing energy generation; this combined strategy is harnessed by the development of a called Plant-Microbial Fuel Cell (PMFC) [82]. In addition, thin-film devices made from nanometer scale protein wires harvested from the microbe *Geobacter sulfurreducens* use the maintained moisture gradient formed within the film when exposed to the humidity [59].

From the aforementioned considerations, although various pinnacles have inspired researchers to harvest solar energy better, it can be noticed that the way such energy is being harvested is fundamentally the same principle as the conventional photovoltaic panels: “cell-like devices” [55].

Other than energy generation from solar, the wind is the second most exploited energy source found in the literature. Renewable energy technologies like solar and wind are the key to reducing emissions in the pathway to net zero, where almost 90% of global electricity generation in 2050 should come from renewable sources, with solar PV and wind together accounting for nearly 70% [24].

Considering nature’s successes, great advancements have been developed to draw energy from wind regarding the conventional wind turbine disadvantages such as noise [61,62], height [63,64,85] and space needed [63,65,85].

To reduce noise, feather and fin shapes are mimicked [61,62]; standard bird feathers’ compilation and movement were employed in the designing of a kinetic building façade with a stage in a grid-like structure, and various feathers per row are allowed to move when hit by air currents. The conceptual design was tested via 3D simulations, obtaining about 2.15 µV with a single row at a maximum 85° full movement [62]. On the other hand, combining owl feathers with the tubercles of humpback whale fins to develop a conceptual airfoil successfully solves aerodynamic noise, but this leads to reduced performance [61].

Moreover, the *Nelumbo nucifera* (sacred Lotus) flower inspired the design of a three-blade wind turbine. After CFD simulations, the experimental results demonstrated an improvement of about 32% compared to NACA 2412 airfoil [63]. Similarly, an increase in performance was obtained by mimicking Maple seed leaf’s shape (also known as “helicopter seeds”) for blades in vertical axis turbines [64].

Instead of enhancing turbine blades’ aerodynamics, the arrangement of several vertical axis wind turbines is studied. For this, mimicking fish schooling helped in increasing energy generation by ten times greater than using standard arrangement, taking advantage of vortices formation while reducing the space needed [65].

Different pinnacles have been accounted for when looking for natural inspiration, where none of the above-mentioned harness wind currents to perform any physiological process. Birds and whales harness stream currents for displacement, while plants help spread pollen. Yet, apart from [62], the system’s design follows the same pattern as the existing wind turbines today, either with a horizontal [63] or vertical axis [61,64,65]. Whilst innovation and enhanced performance significantly set up the wind power harnessing confirmed by real-scale, small-scale experiments or simulation, these studies remained in a concept design proof stage, all reporting mechanical or design limitations that need further experimentation.

Although there have been few significant advancements in the past couple of years [86] in energy generation from waves, several bio-inspired designs can be found to harness waves’ power [87]; these are mostly plants (such as kelp) and elongated organisms (such as centipede, Pelamis, anaconda). Others focus on the human circulatory system. However, recent studies focused on rock crab legs [66] and a combination of the fundamental characteristics of various sea pinnacles [67]. Harnessing wave potential in islands could solve reducing space for either harnessing solar or wind energy. For this, a few design concept ideas can be found in [66] to replace conventional wave power turbine-generator systems installed at the shores. Here, a structure like the rock crab legs was conceptualized to be implemented as a microgrid in coastal areas piled up at the shore [66]. When the fundamental characteristics of several sea pinnacles are combined, a simple structure adaptative device can be developed; a prototype named TALOS is one example, which employs a piezoelectric–electromagnetic device used for the piers of sea-crossing bridges [67]. However, no performance evaluation is given.

A recent study detailed improvements to the environmental adaptability of artificial blades in wind energy collectors through the biomimicry of leaf flutters in nature [68]. The study involved observations and subsequent modeling to determine the palm leaf’s flutter boundary, applying the identified bending and twisting mechanisms to the artificial blade’s design. This bio-inspired blade can initiate energy harvesting at wind speeds as low as 2 m/s, achieving a stable output frequency of 3.56 Hz, and generating a power density of 1.238 μW/cm^3^. Wind tunnel tests confirmed the blade’s efficacy in low-wind conditions, allowing the turbine to operate efficiently and ensure consistent, reliable energy production [68].

Lu et al. engineered a robust, high-strength, multi-compatible metamaterial energy harvesting device aimed at powering continuous, self-sufficient ocean monitoring systems, thereby solving challenges related to power supply and wireless data transmission in marine settings [69]. The device efficiently captures wave energy by exploiting the defective state properties of metamaterials. It boasts a high energy density of 99 W/m^3^ and maintains stable operation across various weather conditions. This performance enables real-time monitoring of ocean parameters and positions the device as superior to conventional solar and wind energy sources in the ocean environment [69].

#### Appendix A.3.3. Energy Storage

Nature has also inspired electricity storage systems when coming to proposals for the reduction in environmental impact. The use of batteries, especially lithium-ion batteries, is the most prominent among electrical storage applications; however, improvements have been proposed through hydrogen batteries or the implementation of more environmentally friendly materials to manufacture the electrodes [88,89]. In this sense, oriented towards creating systems designed to protect the environment, important advances have been made in developing storage systems based on biomimetic strategies. Most advancements are based on the organisms’ ability to store energy [36,80,83] and to enhance battery component materials [71,72,73,89]. However, other problems also need attention. For instance, there is a lack of energy storage technology where no system has the perfect combination of high power, energy density, accessible financial and environmental cost, long cycle and lifespan, easy material availability, and fast response time [36].

Photosynthesis is known for its light-to-energy conversion process, other than its capability to reduce the amount of CO_2_, and for giving glucose as a by-product. To further understand the process and the residual products, an artificial system combining conventional devices, such as PV solar cells, batteries, and hydrogen production modules, among others, connected to replicate the photosynthesis process, was built [70]. It was shown that attention needs to be paid to the performance of each device in the process as well as the link among them to achieve system performance enhancement [70]. However, such a process is normally prolonged due to task management. This inspired the so-called “rewired carbon fixation”, which solves the inefficiency in the photosynthesis process by diversifying tasks within a photosynthetic organism and replacing part of them with modified electroactive microbes, a non-biological equivalent [36].

Wood, as a renewable material, has an anisotropic hierarchical porosity that can provide rapid and low tortuosity pathways for nutrient and water transport. This has inspired scientists to develop different battery components, such as the cathode, the anode, the separator, and the current collectors [71]. Likewise, lignin, a biopolymer abundant in the soil extracted from trees, contains quinone, a conjugated cyclic diketone, which may serve for energy storage through oxide-reduction reactions [72]. This was used in electrodes, but preliminary test results showed a short life cycle, low cyclic efficiency, and high self-discharge rate due to its tendency to degrade in the electrolytes. A solution for this was found by mimicking the Venus flytrap through a reconfigurable graphene cage, effectively solving the cyclic lifetime and resulting in an economical and renewable material [72]. Further investigations to enhance electrode characteristics were performed. Sucrose, as an organic carbon source, was used to improve the electrical conductivity of the anode in lithium-based batteries, reducing the charge transfer resistance and benefiting the charge-discharge cycle [73]. Another category that pertains to the biomimetic is the one of edible batteries, which are able to exploit the energy contained in polysaccharides to allow them to be ingested to perform some medical controls [74]. More recently, this concept has been developed by immobilizing quercetin and riboflavin on active carbons obtained from coconuts [90].

A recent investigation introduced biomimetically optimized vertically aligned annular fins to enhance latent heat thermal energy storage (LHTES) systems [75]. The topological optimization resulted in fins whose fractal dimensions mirror the structure of plant leaf veins. Similarly to how leaf veins optimize the transport of nutrients and heat in nature, these fins improve effective heat transfer within LHTES. Compared to conventional fin designs, these topology-optimized fins reduce the phase change material’s melting time by a range of 45.9% to 18.4%, which facilitates faster thermal energy storage rates, achieves higher temperature uniformity, and boosts heat transfer efficiency. This bio-inspired methodology is supported by both numerical simulations and experimental validations, positioning it as a promising advancement for thermal energy storage [75].

Furthermore, another recent study successfully simulated the internal structure of a conch shell to develop a biomimetic conch phase change capsule [76]. This model precisely imitates the spiral fins found inside the conch, resulting in a substantial increase in the heat exchange surface area. This design modification dramatically improved the thermal energy storage efficiency by 402.89% [76].

#### Appendix A.3.4. Energy Management Optimization

Many organisms’ behavior has inspired the development of alternative algorithms to spend minimal resources to execute processes more efficiently [77,91,92]. When looking for food, the *Physarum polycephalum*’s and ants’ paths represent greater communication efficiency [77,91]. In particular, the slime mold *Physarum polycephalum* is a unicellular creature, yet growing up to considerable dimensions, such as thousands of centimeters. Its behavior and growth are characteristic because when food sustenance is scarce, it spreads and grows in search of food, organizing itself into a network of tubular structures that connect quickly and efficiently. It grows faster in the dark than in illuminated areas [77]. In contrast, to forage for food, ants initially wander randomly. Upon finding a food source, they return to their anthill, leaving behind a small amount of pheromone along the way. When other ants find this compound, they will follow the trail. If they find food, they will reinforce the trail with more pheromones until they return to the colony. In the case of a short route, the pheromone density will be high, making it more likely that other ants will follow this path. As a consequence, there will be positive feedback that leads all ants to follow a single path. This mechanism has the advantage of avoiding approaching a solution that is only locally suitable since, as the number of ants on the path to the food source increases, the pheromone concentration will become stronger, replenishing as fast as it evaporates [91].

In the mobility and transportation strategy, we can mention the case of Japan, where a study was conducted on a protozoan using the *Physarum polycephalum* mold, in which the efficient way in which this mold creates its networks to find food was investigated [77]. In that study, use was made of a translucent plate that replicated the geographical features of Tokyo and its surroundings. The terrain’s elevations and the coastline’s contour were mimicked by placing points of light. Food sources were placed at different strategic locations in the city. As a result, a network of tubules created by the mold was obtained very similarly to the existing railway network, which has been optimized and improved over the years. In [91], the problem presented in La Paz and El Alto in Bolivia was addressed, where the major issue is transportation organization linked to vehicular congestion. In order to optimize the existing design, it was proposed to address two aspects: the combinatorial optimization of flow patterns in metropolitan transportation and the establishment of collaborative public policies on transportation. Modeling work was carried out for the combinatorial optimization of transport flow patterns by applying the ant colony algorithm and Dijkstra’s algorithm (minimum paths algorithm). The mold *Physarum polycephalum* was also considered for studies performed approximating the highways of Mexico and the Iberian Peninsula [78,79].

A recent study examined the theoretical application of bio-inspired algorithms, specifically the Particle Swarm Optimization (PSO) algorithm and the Genetic Algorithm (GA), to enhance the energy availability of a renewable energy system designed for an existing university building in a tropical climate. The research followed a multi-step process focusing on designing the system to fit space constraints and optimizing its overall energy management [80]. The initial optimization, which used the PSO algorithm to determine the ideal combination of power generators within the available space, resulted in a 10% increase in the energy deficit. This finding underscores the complex challenges inherent in integrating comprehensive renewable energy systems into existing buildings with restrictive parameters. However, an independent application of the PSO algorithm to optimize the discharge management of the battery bank demonstrated a positive outcome, achieving a 2% efficiency improvement when incorporated into the original, pre-optimization system. The overall findings showcase the potential of algorithms like PSO and GA for specific, targeted optimization tasks within renewable energy systems, warranting further research to refine their tailored application for performance enhancement in existing infrastructure [80].
